# Bacteria and Protozoa Differentially Modulate the Expression of Rab Proteins

**DOI:** 10.1371/journal.pone.0039858

**Published:** 2012-07-20

**Authors:** Elsa Seixas, José S. Ramalho, Luís J. Mota, Duarte C. Barral, Miguel C. Seabra

**Affiliations:** 1 CEDOC, Faculdade de Ciências Médicas (FCM), Universidade Nova de Lisboa, Lisboa, Portugal; 2 Instituto Gulbenkian de Ciência, Oeiras, Portugal; 3 Instituto de Tecnologia Química e Biológica, Universidade Nova de Lisboa, Oeiras, Portugal; BioScience Project, United States of America

## Abstract

Phagocytic cells represent an important line of innate defense against microorganisms. Uptake of microorganisms by these cells involves the formation of a phagosome that matures by fusing with endocytic compartments, resulting in killing of the enclosed microbe. Small GTPases of the Rab family are key regulators of vesicular trafficking in the endocytic pathway. Intracellular pathogens can interfere with the function of these proteins in order to subvert host immune responses. However, it is unknown if this subversion can be achieved through the modulation of Rab gene expression. We compared the expression level of 23 distinct Rab GTPases in mouse macrophages after infection with the protozoan *Plasmodium berghei*, and the bacteria *Escherichia coli* and *Salmonella enterica*. We found that *P*. *berghei* induces an increase in the expression of a different set of Rab genes than *E*. *coli* and *S*. *enterica*, which behaved similarly. Strikingly, when one of the Rab proteins whose expression was increased by *P*. *berghei*, namely Rab14, was silenced, we observed a significant increase in the phagocytosis of *P*. *berghei*, whereas Rab14 overexpression led to a decrease in phagocytosis. This suggests that the parasite might induce the increase of Rab14 expression for its own advantage. Similarly, when Rab9a, whose expression was increased by *E*. *coli* and *S*. *enterica*, was silenced, we observed an increase in the phagocytosis of both bacterial species, whereas Rab9a overexpression caused a reduction in phagocytosis. This further suggests that the modulation of Rab gene expression could represent a mechanism of immune evasion. Thus, our study analyzes the modulation of Rab gene expression induced by bacteria and protozoa and suggests that this modulation could be necessary for the success of microbial infection.

## Introduction

Infection by intracellular pathogens remains a major cause of human morbidity and mortality worldwide. Phagocytosis is an important process of host defense against invading microorganisms that involves their binding to the cell surface, internalization and subsequent targeting to lysosomes for degradation. The uptake of pathogens and the activation of membrane trafficking pathways that lead to microbial killing and degradation are key to an efficient host defense, since they are also necessary to elicit an immune response [1]. Pathogens that are internalized by phagocytosis are sequestered in compartments originating from the plasma membrane, termed phagosomes. Newly formed phagosomes are unable to kill or degrade their content and must therefore engage in a complex maturation process [2], [3].

*Plasmodium berghei* is an intracellular protozoan parasite that infects and replicates intracellularly in two main cell types, hepatocytes and erythrocytes, causing malaria [4], [5]. *Escherichia coli* is a bacterium commonly found in the lower intestine. Most strains are non-pathogenic and belong to the normal gut flora of humans, but some serotypes can cause food poisoning in humans. However, if the bacteria get access to niches outside the gut, they can cause potentially deadly infections such as urinary tract infections, meningitis or even septicemia [6], [7]. *Salmonella enterica* serovar Typhimurium (*S*. Typhimurium) typically causes gastroenteritis in humans. These bacteria are facultative intracellular pathogens that can infect a wide variety of host cell types.

Phagocytic cells are able to eliminate pathogens through a sequential phagosomal maturation process, which involves fusion with different compartments of the endocytic pathway [8], [9]. During maturation, phagosomes fuse with lysosomes forming a phagolysosome and acquire degradative and microbicidal properties, leading to the destruction of internalized pathogens. Internalization, phagosome maturation and trafficking are regulated by small GTPases of the Rab family [10], [11], [12]. Several reports have highlighted the role of these proteins in the interaction of microbial pathogens with different host cell types. There are over 70 Rab proteins identified in mammalian cells [13], [14] and more than 20 can associate with phagosomal membranes [15], [16], [17]. However, few of these have been studied. Rab5 becomes associated with the phagosome immediately after phagocytosis and it was shown to be involved in the efficient elimination of *Leishmania donovani* [18]. Further, deficient recruitment of Rab7, which regulates trafficking to late endosomes, has been associated with inhibition of phagosome maturation by *L*. *donovani* [19]. In the case of mycobacteria, previous work has shown that a blockade in phagosome maturation occurs between the steps regulated by Rab5 and Rab7, *i*.*e*. between early and late endosomes [20]. Rab14 also plays a role in mycobacterial infections, since it is reported to be mainly localized to early endosomes and to contribute to the arrest of mycobacterial phagosomes [21]. Finally, compared to a model phagosome, *S*. Typhimurium-containing phagosomes have an excess of some Rabs (5a, 5b, 5c, 7a, 11a and 11b) and are devoid of others (Rabs 8b, 13, 23, 32 and 35) [15]. It is thought that through this selective recruitment of Rabs, *Salmonella* inhibits the fusion of its phagosome with lysosomes. However, other studies indicate that *Salmonella* phagosomes fuse extensively with lysosomes [22]. Despite this knowledge about how pathogens interfere with Rab GTPase recruitment and activity, it is largely unknown if pathogens can modulate the expression of the genes encoding these proteins to interfere with phagocytosis and phagosomal maturation. In this study, we investigated the effect of different pathogens on the expression of 23 Rab GTPases by mouse macrophages. For this, we used the malaria parasite, *P*. *berghei*, and non-pathogenic *E*. *coli* and pathogenic *S*. Typhimurium Gram-negative bacteria. We observed a significant variability in the pool of Rab GTPases that have their expression changed upon phagocytosis of the malaria parasite or bacteria. Further, we demonstrate that Rab14 is involved in phagocytosis of *P*. *berghei*, but has no role on the phagocytosis of bacteria. In contrast, Rab9a is involved in the phagocytosis of *E*. *coli* and *S*. *enterica* but not in the phagocytosis of *P*. *berghei*. Thus, our results suggest that specific Rabs are modulated by different pathogens. Furthermore, we found evidence for a role of Rab14 and Rab9a in the phagocytosis of *P*. *berghei* and *E*. *coli*/*S*. *enterica*, respectively, and propose that the parasite and the bacteria can interfere with the expression of Rab GTPases to escape ingestion by macrophages.

**Figure 1:**
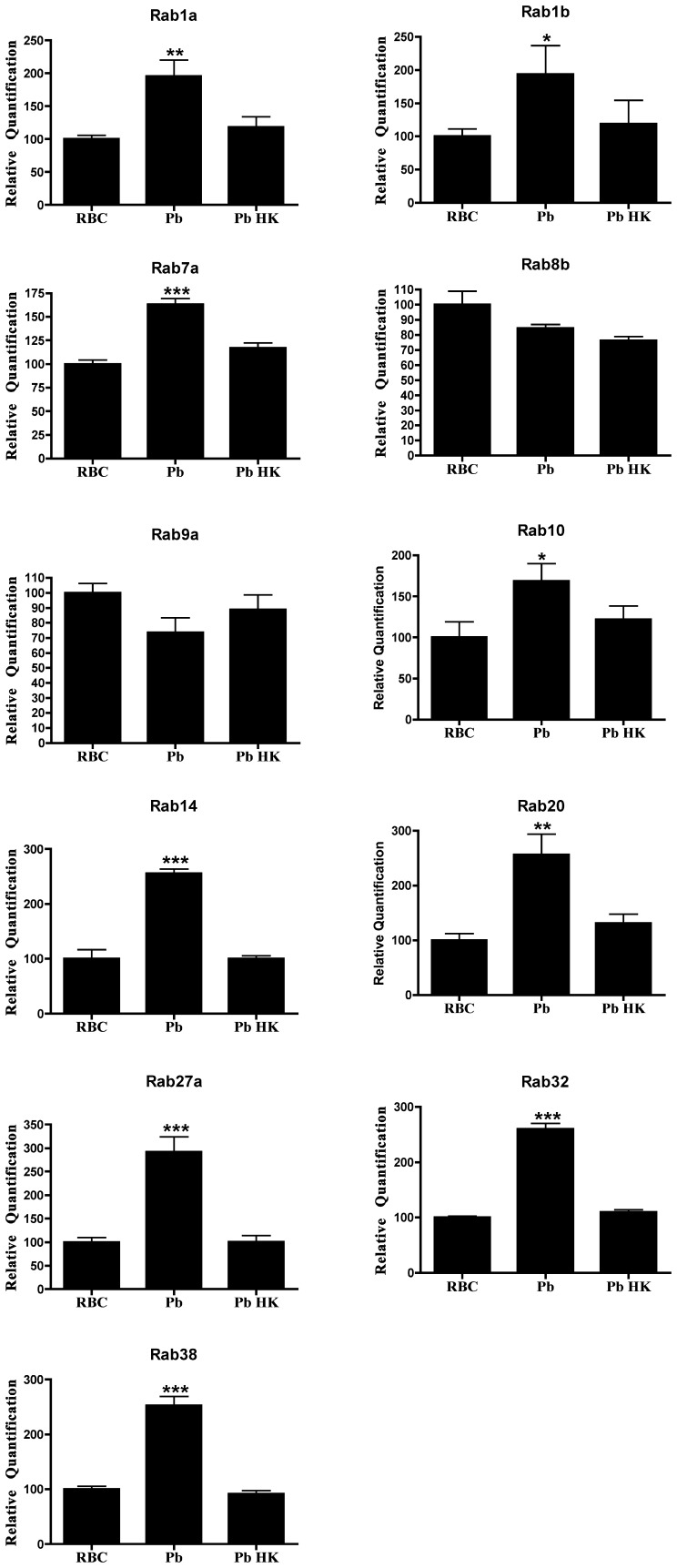
Expression of Rab GTPase genes on primary macrophages after infection with *P*. *berghei*. Columns represent the relative quantification of the mRNA levels of each Rab GTPase, analyzed by real-time quantitative PCR, after reverse transcription of total mRNA and normalized against GAPDH gene expression levels. Error bars indicate the standard error of the mean of 5 independent experiments. Statistical significance (*p<0.05, **p<0.01, ***p<0.001) refers to the difference between macrophages incubated with infected red blood cells (*Pb*) or with heat-killed parasites (*Pb* HK) and macrophages incubated with uninfected red blood cells (RBC).

**Table 1:** Rab GTPase gene expression levels in macrophages infected with *P*. *berghei* (Pb), *E*. *coli* (Ecoli), *S*. Typhimurium (Salmo), LPS or latex beads (Beads).

Rab	Pb	Ecoli	Salmo	LPS	Beads
**1a**	↑			↑	
**1b**	↑			↑	
**4b**				↑	
**5a**				↑	
**5b**				↑	
**5c**				↑	
**7a**	↑			↑	
**8a**				↑	
**8b**		↑	↑	↑	
**9a**		↑	↑	↑	
**10**	↑	↑	↑	↑	↑
**11a**				↑	
**11b**				↑	
**14**	↑			↑	
**20**	↑	↑	↑	↑	
**22b**				↑	
**27a**	↑				
**32**	↑	*	*	↑	
**34**				↑	
**38**	↑	*	*	↑	
**39a**				↑	
**39b**				↑	
**43**				↑	

Results of the expression levels of distinct Rab GTPases obtained by RT-qPCR are summarized in this table. An increase in Rab GTPase expression level above 1.5 fold is represented by an arrow (↑), while no difference in expression level, as compared to macrophages incubated with uninfected red blood cells or medium alone, is represented by an empty square. (*) Rab32 and Rab38 have their expression increased only when macrophages are infected with heat-killed bacteria and not with live bacteria.

## Materials and Methods

### Ethics Statement

Mice were bred and maintained under specific pathogen-free (SPF) conditions, according to protocols approved by local (Instituto Gulbenkian de Ciência) and national (Portuguese Official Veterinary Department; Direcção Geral de Veterinária) ethics committees. Mouse experimental protocols were approved by the Instituto Gulbenkian de Ciência Ethics Committee and the Portuguese Veterinary General Division.

### Mice and Parasites

C57BL/6 and Balb/c mice were infected intraperitoneally (i.p.) with 10^5^ infected red blood cells of *Plasmodium berghei* ANKA, *Plasmodium berghei* ANKA-GFP or *Plasmodium berghei* ANKA-RFP strains. Parasitemia was monitored by Giemsa-stained blood smears or by flow cytometry in the case of the GFP and RFP parasites.

**Figure 2:**
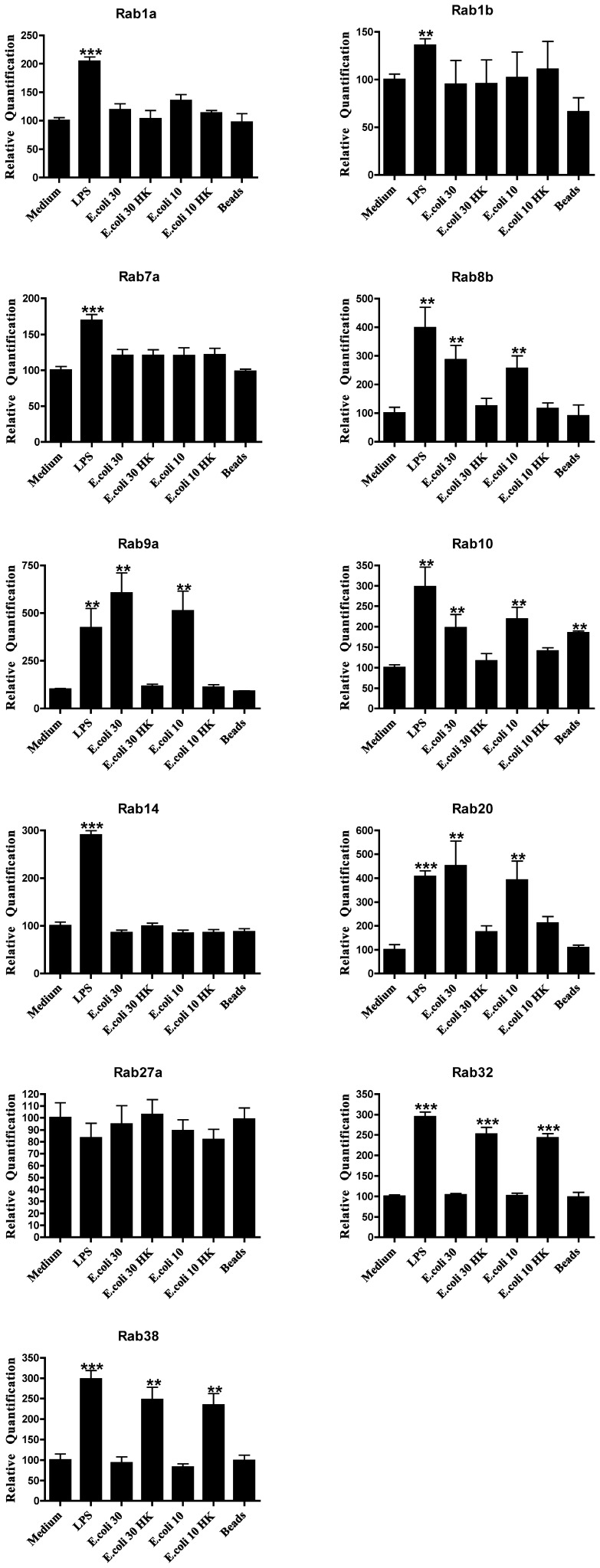
Expression of Rab GTPase genes on primary macrophages after infection with *E*. *coli*. Columns represent the relative quantification of the mRNA levels of each Rab GTPase, analyzed by real-time quantitative PCR, after reverse transcription of total mRNA and normalized against GAPDH gene expression levels. Macrophages were incubated with live or dead (HK) bacteria at a multiplicity of infection (MOI) of 30∶1 (*E*. *coli* 30, *E*. *coli* 30 HK) and 10∶1 (*E*. *coli* 10, *E*. *coli* 10 HK). Cultures of macrophages with medium only (Medium) and cultures with LPS (1 µg/ml) or latex beads (beads) were also performed. Error bars indicate the standard error of the mean of 5 independent experiments. Statistical significance (**p<0.01, ***p<0.001) refers to the difference between macrophages incubated with live or dead bacteria or incubated with LPS or latex beads and macrophages incubated with medium alone.

**Figure 3:**
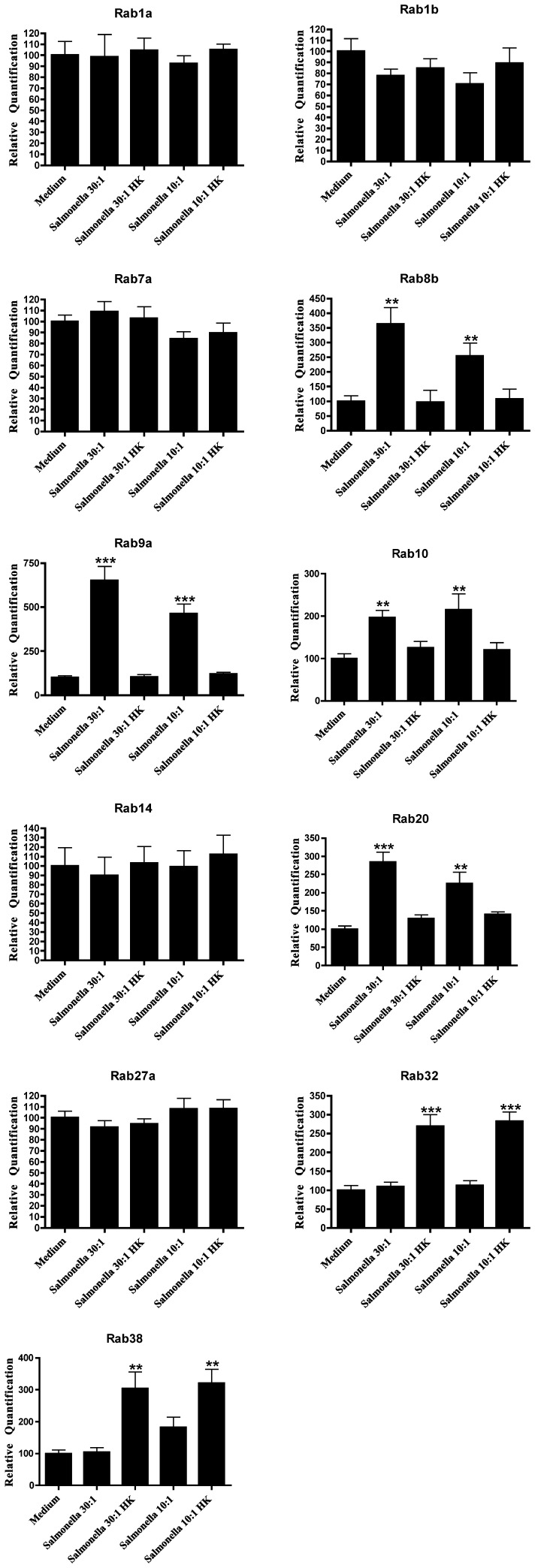
Expression of Rab GTPase genes on primary macrophages after infection with *Salmonella*. Columns represent the relative quantification of the mRNA levels of each Rab GTPase, analyzed by real-time quantitative PCR, after reverse transcription of total mRNA and normalized against GAPDH gene expression levels. Macrophages were incubated with live or dead (HK) bacteria at MOI of 30∶1 (Salmonella 30, Salmonella 30 HK) and 10∶1 (*Salmonella* 10, *Salmonella* 10 HK). Error bars indicate the standard error of the mean of 3 independent experiments. Statistical significance (**p<0.01, ***p<0.001) refers to the difference between macrophages incubated with live or dead bacteria and macrophages incubated with medium alone.

### Culture and Purification of Parasite Schizonts

Infected mice at day 5 or 6 after infection were bled and the blood used for *in vitro* culture for 18–20 h so that parasites could develop into schizonts. This is achieved after overnight culture at 37°C in RPMI medium containing FBS and gassed with a mixture of 10% CO_2_, 5% O_2_ and 85% N_2_. Schizonts were enriched by magnetic isolation as described previously [23]. In all experiments purity was greater than 90%.

**Figure 4:**
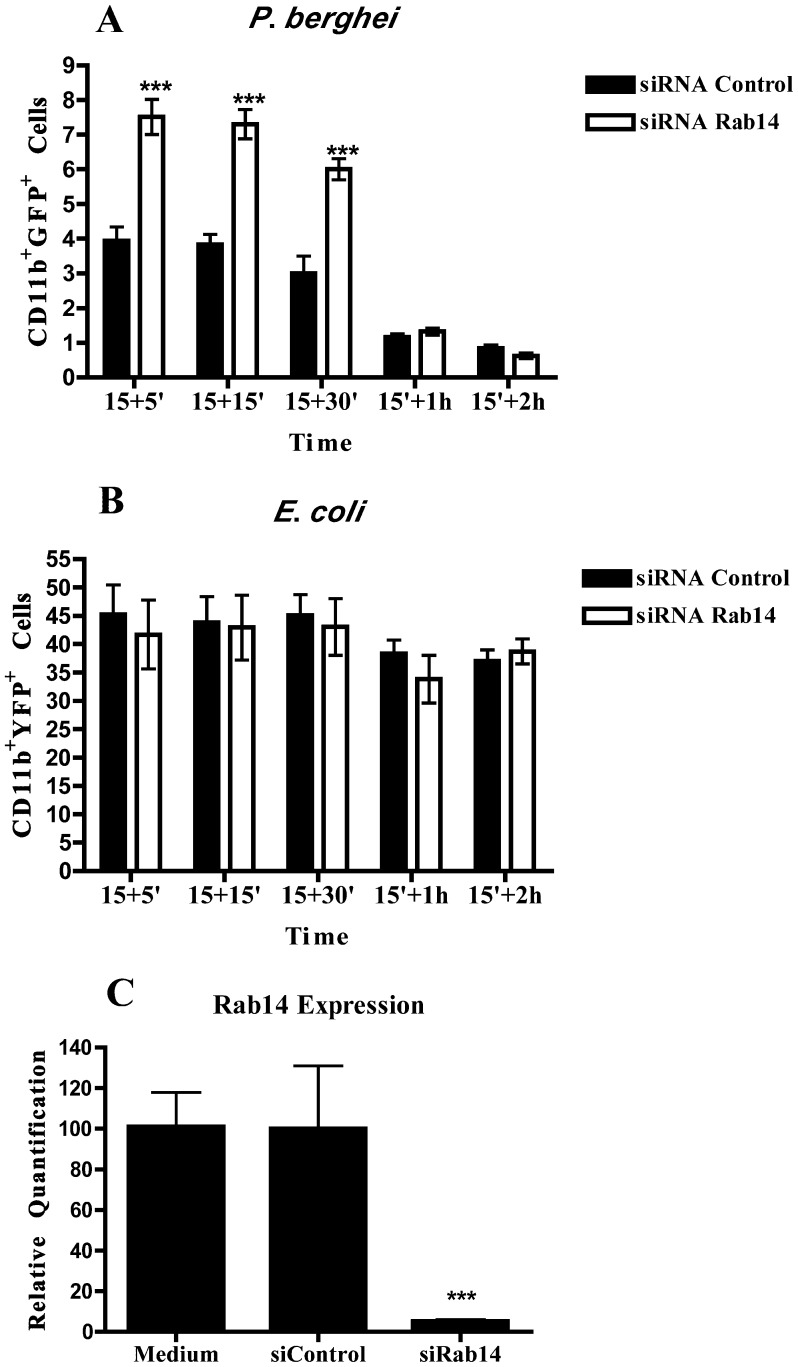
Rab14 silencing by siRNA increases phagocytosis of *P*. *berghei*. Macrophages were transfected with siRNA for Rab14 or siRNA control for 48 h, and infected with YFP-*E*. *coli* or GFP-*P*. *berghei*. After 15 minutes of incubation, cells were washed, chased for different time points and analyzed by flow cytometry. (**A**) Columns represent the percentage of cells positive for CD11b and GFP analyzed after incubation with *P*. *berghei*. (**B**) Columns represent the percentage of cells positive for CD11b and YFP analyzed after incubation with *E*. *coli*. (**C**) Efficiency of Rab14 silencing in macrophages treated with siRNA for Rab14, with siRNA control or not treated. Error bars indicate the standard error of the mean of 4 independent assays. Statistical significance (***p<0.001) refers to the difference between macrophages treated with siRNA for Rab14 and siRNA control.

### Bacterial Strains and Plasmids

*E*. *coli* M61655 K12 strain and *S*. Typhimurium (strain NCTC 12023) were inoculated in Luria Bertani broth with the relevant antibiotics and incubated overnight at 37°C with vigorous shaking. For fluorescence microscopy and flow cytometry experiments, *E*. *coli* harbouring a plasmid encoding YFP (*E*. *coli*-YFP) or CFP (*E*. *coli*-CFP), and *S*. Typhimurium harbouring pDsRed (DsRed-*Salmonella*) [24] were used. Bacteria were counted by flow cytometry using 3 µm beads.

**Figure 5:**
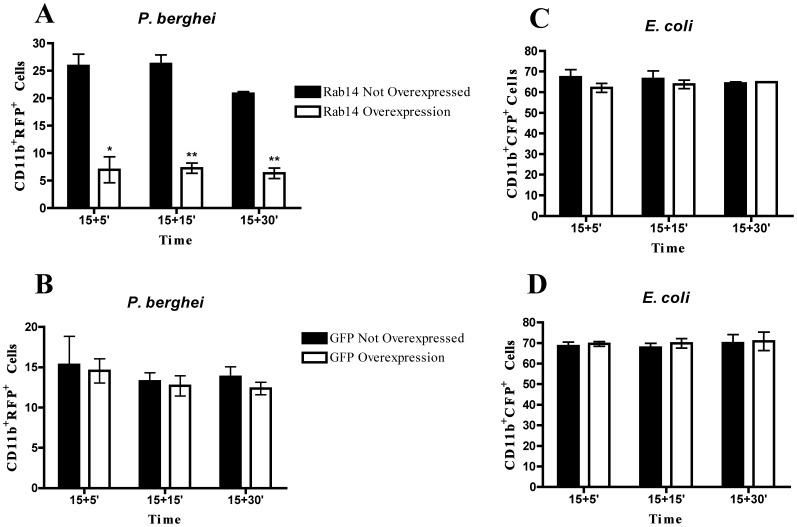
Rab14 overexpression decreases phagocytosis of the malaria parasite. Macrophages were transfected with GFP-Rab14 DNA or GFP as a control for 8 h and then infected with RFP-*P*. *berghei* or CFP-*E*. *coli*. After 15 minutes of incubation, cells were washed and chased for different time points and analyzed by flow cytometry. (**A**) Columns represent the percentage of GFP-Rab14 overexpressing cells or cells that do not overexpress Rab14 with internalized RFP-*P*. *berghei*. (**B**) Columns represent the percentage of GFP overexpressing cells or cells that do not overexpress GFP with internalized RFP-*P*. *berghei*. (**C**) Columns represent the percentage of Rab14-GFP overexpressing cells or cells that do not overexpress Rab14 with internalized CFP-*E*. *coli*. (**D**) Columns represent the percentage of GFP overexpressing cells or cells that do not overexpress GFP with internalized CFP-*E*. *coli*. Error bars indicate the standard error of the mean of 3 independent assays. Statistical significance (*p<0.05, **p<0.01) refers to the difference between macrophages that do not overexpress Rab14 and macrophages that overexpress Rab14.

**Figure 6:**
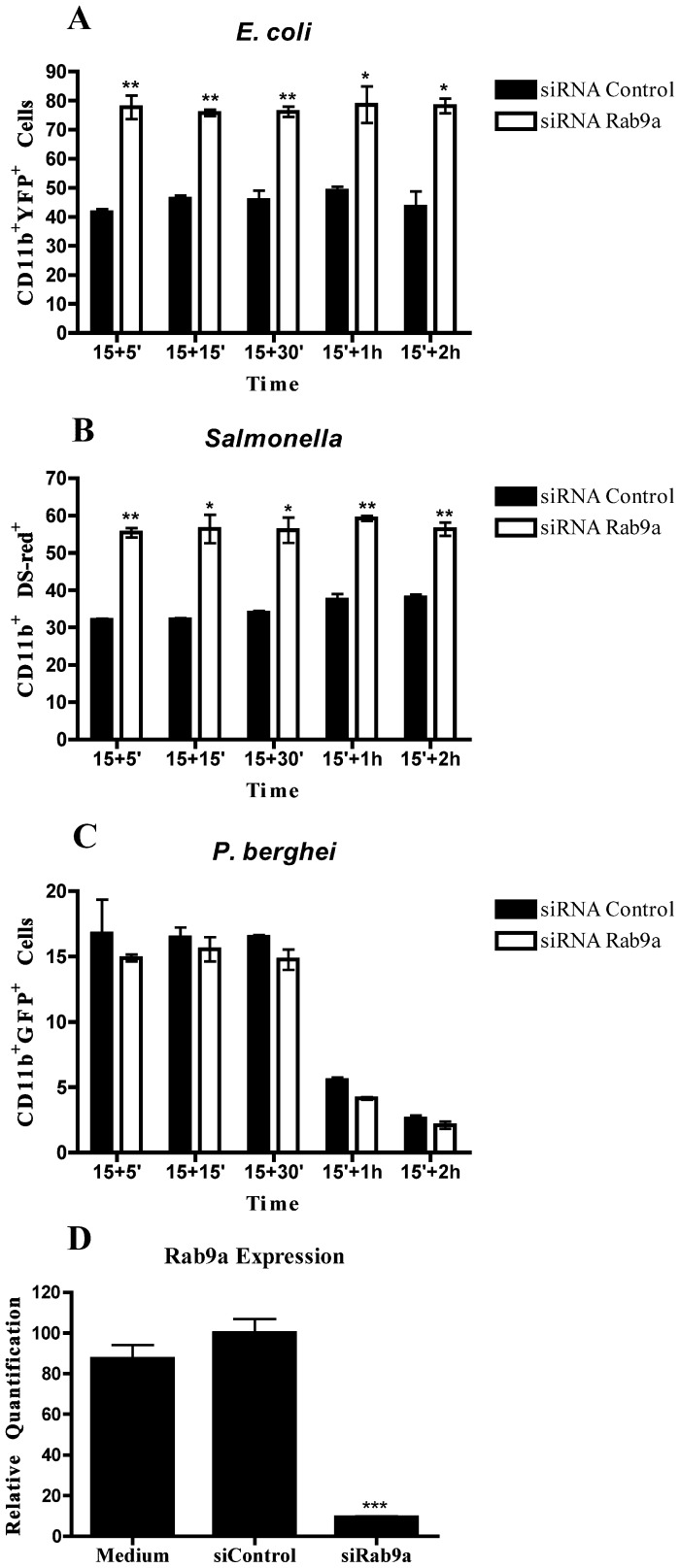
Rab9a silencing by siRNA increases phagocytosis of *E*. *coli* and *Salmonella*. Macrophages were transfected with siRNA for Rab9a or siRNA control for 48 h, and infected with YFP-*E*. *coli*, DS-red-*Salmonella* or GFP-*P*. *berghei*. After 15 minutes of incubation, cells were washed, chased for different time points and analyzed by flow cytometry. (**A**) Columns represent the percentage of cells positive for CD11b and YFP analyzed after incubation with *E*. *coli*. (**B**) Columns represent the percentage of cells positive for CD11b and DsRed analyzed after incubation with *Salmonella* (**C**) Columns represent the percentage of cells positive for CD11b and GFP analyzed after incubation with *P*. *berghei*. (**D**) Efficiency of Rab9a silencing in macrophages treated with siRNA for Rab9a, with siRNA control or not treated. Error bars indicate the standard error of the mean of 4 independent assays. Statistical significance (*p<0.05, **p<0.01) refers to the difference between macrophages treated with siRNA for Rab9a and siRNA control.

**Figure 7:**
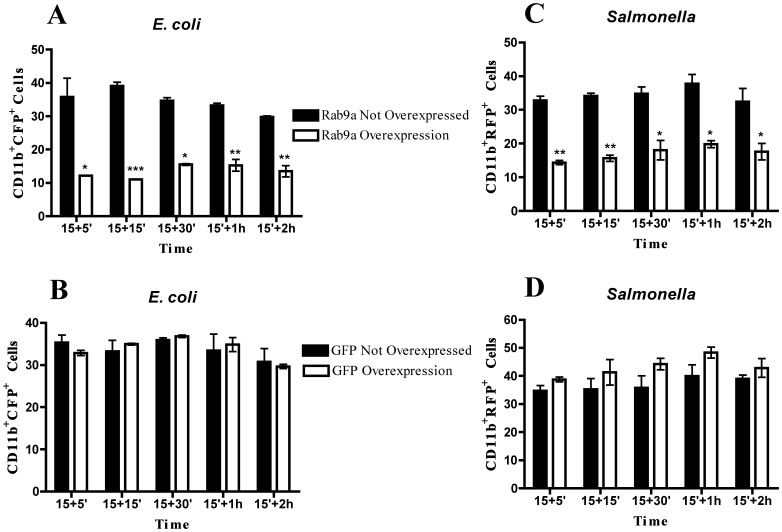
Rab9a overexpression decreases phagocytosis of *E*. *coli* and *Salmonella*. Macrophages were transfected with GFP-Rab9a or GFP for 8 h and then infected with CFP-*E*. *coli* or DsRed-*Salmonella*. After 15 minutes of incubation, cells were washed and chased for different time points and analyzed by flow cytometry. (**A**) Columns represent the percentage of GFP-Rab9a overexpressing cells or cells that do not overexpress Rab9a with internalized CFP-*E*. *coli*. (**B**) Columns represent the percentage of GFP overexpressing cells or cells that do not overexpress GFP with internalized CFP-*E*. *coli*. (**C**) Columns represent the percentage of Rab9a-GFP overexpressing cells or cells that do not overexpress Rab9a with internalized DsRed-*Salmonella* (**D**) Columns represent the percentage of GFP overexpressing cells or cells that do not overexpress GFP with internalized DsRed-*Salmonella*. Error bars indicate the standard error of the mean of 2 independent assays. Statistical significance (*p<0.05, **p<0.01, ***p<0.001) refers to the difference between macrophages that do not overexpress Rab9a and macrophages that overexpress Rab9a.

**Figure 8:**
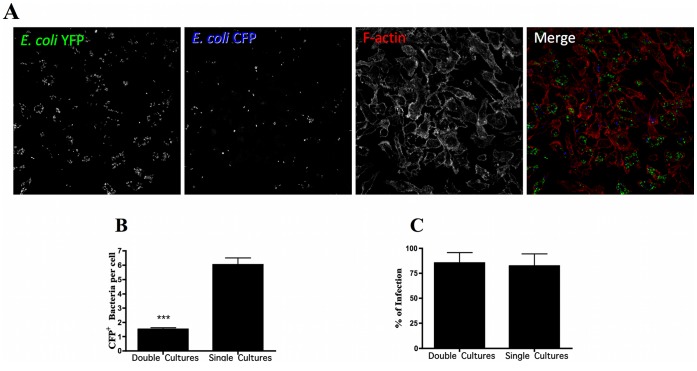
Macrophages are attenuated for secondary phagocytosis. Macrophages were challenged with two rounds of infection using *E*. *coli* labeled with YFP and CFP. Cells were infected with *E*. *coli*-YFP and two hours later challenged with CFP-labeled bacteria for 15 minutes. (**A**) Representative pictures showing macrophages infected with *E*. *coli*-YFP (in green) and *E*. *coli*-CFP (in blue) analyzed by confocal laser scanning microscopy. Actin cytoskeleton was stained with Phalloidin Alexa Fluor 568 (in red). (**B**) Number of CFP^+^ bacteria per cell, determined microscopically in cultures infected with both *E*. *coli*-YFP and *E*. *coli*-CFP (Double cultures) and cultures infected only with *E*. *coli*-CFP (Single cultures), is represented (n = 500). (**C**) The percentage of infection determined microscopically in cultures infected with both *E*. *coli*-YFP and *E*. *coli*-CFP (Double cultures) and cultures infected only with *E*. *coli*-CFP (Single cultures) is shown (n = 400). Statistical significance (***p<0.001) refers to the difference between double and single cultures.

### Isolation of Primary Macrophages

Cells were collected from the bone marrow of mice and differentiated *in vitro* for 8 days in Iscove’s medium supplemented with 10% Fetal Bovine Serum (FBS), 0.5 mM sodium pyruvate, 100 units/mL of penicillin, 100 mg/mL streptomycin, 5×10^−5^ M 2-mercaptoethanol and 30% L929-cell conditioned medium. Cells were harvested and stained for CD11b and F4/80 to determine the purity of the population. In all experiments the purity was greater than 90%.

### Macrophage and Pathogen Cultures

Primary macrophages were counted and plated in 24 well plates at 5×10^5^ cells per well. *Plasmodium*-infected erythrocytes were added at a ratio of 30∶1 (iRBC:Macrophage), the cultures incubated for 15 min., washed with medium to eliminate the parasites that were not internalized, and incubated for 4 hours, after which samples were frozen for RT-PCR analysis. For microscopy and flow cytometry, cultures were incubated after washing, for a further 5, 15, 30 min. or 1 h and 2 h. Bacteria were added to the macrophages at a ratio of 30∶1 or 10∶1 and the incubation followed the same protocol as for *Plasmodium*. To obtain heat-killed bacteria or *Plasmodium*, preparations were incubated at 95°C for 15 minutes. Uninfected erythrocytes were used as a negative control. Beads (3 µm) were added to the cultures at a ratio of 30∶1 and the same protocol was followed. LPS was used at 1 µg/ml as a positive control.

### Flow Cytometry

Cells were incubated with Cy5-conjugated anti-CD11b diluted in PBS containing 2% FBS and 0.01% NaN_3_. Data were acquired and analyzed on a FACScalibur using CellQuest software (Becton Dickinson).

### Confocal Laser Scanning Microscopy

Macrophages were transfected or not with a plasmid encoding GFP-Rab14, allowed to adhere to coverslips and infected with bacteria or the malaria parasite for the time points studied. Cells were then fixed with 2% paraformaldehyde, stained with Phalloidin Alexa Fluor 568 (Molecular Probes-Invitrogen) and the coverslips mounted with Mowiol. Images were acquired with a Zeiss LSM710 confocal microscope using a 63× objective.

### Real-time Quantitative PCR

Total RNA was extracted using the RNeasy Mini Kit (Qiagen). 1 µg of total RNA was reverse-transcribed using SuperScriptII RNase H-reverse transcriptase (Invitrogen, CA) and random hexamer primers (Invitrogen). Reactions were incubated at 65°C for 5 min, then at 25°C for 10 min, followed by 42°C for 50 min and finally at 70°C for 15 min. Real-time quantitative PCR (RT-qPCR) was performed in ABI Prism 7900HT system using ABI Power SYBR Green PCR Master Mix. The list of RT-qPCR primers used is shown in Table S1. The messenger RNA (mRNA) levels were normalized against Glyceraldehyde-3-phosphate dehydrogenase (GADPH).

### Rab14 and Rab9a siRNA Silencing and Overexpression

Rab14 and Rab9a silencing were achieved using siGENOME SMARTpool (Dharmacon) specific for *Mus musculus* Rab14 or Rab9a. The list of siRNA sequences is shown in Table S2. Control siRNA was done with nontargeting siRNA pool (Dharmacon). Primary macrophages were transfected with 2 µg of siRNA in a nucleoporator buffer supplied by the manufacturer (Amaxa Biosystems). Cells were nucleoporated according to the manufacturer’s protocol. The cells were then plated and incubated for 48 h prior to bacterial or parasite infections. Rab14 and Rab9a overexpression was performed using the pENTR GFP C2 mRab14 or mRab9a constructs and pENTR GFP as a control. In order to generate pENTR-GFP-Rab9a and Rab14, a Gateway® (Invitrogen) and mammalian expression vector was used. pENTR-GFPC1 and C2 were generated from pENTR-V5 [25], by swapping part of the CMV promoter, V5 tag and the polylinker with the equivalent sequences containing GFP sequence from pEGFPC1 and C2 (Clontech) respectively, using NdeI/BamHI restriction sites. Rab9a murine coding sequence and part of 3′ UTR were produced by RT-PCR amplification (forward primer-atcactcgagaaatggcaggaaaatcgtctct, reverse primer-aagtggtaccccatttccttgtgggtca), digested with *Xho*I/*Kpn*I and cloned into pENTR-GFPC1 with the same restriction enzymes. Rab14 murine coding sequence and part of 3′ UTR were produced by RT-PCR amplification (forward primer-agcaaccccccagtgaattcatggcaactgcaccg, reverse primer-gcaggtcgacgtactgcttccaacagacagagg) using total RNA isolated from AtT20 cell line as a template, digested with *Eco*RI/*Sal*I and cloned into pENTR-GFPC2 with the same restriction enzymes. Primary macrophages were transfected with 5 µg DNA as described above, plated and allowed to express the construct for 6 to 8 hours prior to infection.

### Statistical Analysis

Experimental data were analyzed using the GraphPad Prism statistical analysis package (GraphPad Software, Inc., USA). Statistical differences were analyzed using Student’s t-test. A P value <0.05 was considered statistically significant.

## Results

### *P*. *berghei* Infection Upregulates the Expression Level of Specific Rab Genes

We investigated the effect of infection of macrophages on the expression of 23 different Rab GTPases. To study the effect of *P*. *berghei* infection on the expression levels of different Rabs, we cultured macrophages in the presence of parasite-infected erythrocytes, and then measured gene expression by real-time quantitative PCR (RT-qPCR). We investigated 23 distinct Rab GTPases whose expression had been confirmed in primary macrophages [26] and that were linked to phagocytosis or the endocytic pathway. We used uninfected red blood cells (RBC) as a negative control and heat-killed parasite-infected red blood cells (Pb HK) to determine if there were any differences between live (Pb) and dead parasites (Fig. 1). The results showed that, compared to the negative control, the live malaria parasite was able to induce an increase in the expression of Rab1a, Rab1b, Rab7a, Rab10, Rab14, Rab20, Rab27a, Rab32 and Rab38 (Fig. 1). Moreover, heat-killed parasites showed the same expression levels of the different Rabs as the negative control, indicating that the differences detected with live parasites are specific (Fig. 1). Furthermore, the other Rabs investigated showed no significant differences in expression (Table 1).

### *E*. *coli* and *S*. Typhimurium Infection Upregulate the Expression Level of Specific Rab Genes

We next investigated if infection of macrophages with a non-pathogenic strain of *E*. *coli* had a similar effect on the expression of the Rab GTPases studied. We used a multiplicity of infection (MOI) of 10∶1 that has been shown to be able to activate macrophages and also 30∶1, since this was the ratio used for *P*. *berghei* infection. For both MOIs we also used heat-killed bacteria, to determine if there were any differences between live and dead bacteria. Furthermore, latex beads were used as an inert stimulus, since they can be internalized by macrophages without subverting the phagocytic pathway. Finally, we used lipopolysaccharide (LPS) as a positive control as it is a potent activation stimulus for macrophages, and cells incubated with medium as a negative control. In contrast with *P*. *berghei*, live *E*. *coli* induced an increase in the expression of Rab8b, Rab9a, Rab10 and Rab20, compared to the negative control (Fig. 2). Surprisingly, heat-killed bacteria induced an increase in the expression of Rab32 and Rab38, while the live bacteria had no effect on the expression of these two Rabs (Fig. 2). All the other Rab GTPases studied showed no significant differences as compared to medium alone (Table 1). Strikingly, LPS induced the increase in expression of all the Rabs studied, except for Rab27a while the latex beads only showed a significant difference for Rab10, when compared to medium alone (Fig. 2). To compare the effects on Rab expression of non-pathogenic *vs*. pathogenic bacteria, we used a Gram-negative pathogenic bacterium (*S*. Typhimurium). The results obtained with *S*. Typhimurium were identical to *E*. *coli* (Fig. 3), since we also detected an increase in the expression levels of Rab8b, Rab9a, Rab10 and Rab20 when compared to medium alone. Moreover, Rab32 and Rab38 also showed an increase in expression with heat-killed *S*. Typhimurium (Fig. 3).

The differences in expression of Rab GTPases upon infection of macrophages with different microorganisms are summarized in Table 1. While *P*. *berghei* induced an increase in the expression of nine different Rab GTPases, namely Rab1a, Rab1b, Rab7a, Rab10, Rab14, Rab20, Rab27a, Rab32 and Rab38, both *E*. *coli* and *S*. Typhimurium led to an increase in the expression of four different Rab GTPases, namely Rab8b, Rab9a, Rab10 and Rab20. Therefore, the only two Rab GTPases that had their expression increased after incubation with both bacteria and the malaria parasite were Rab10 and Rab20. Thus, a significantly different pool of Rab GTPases has its expression altered upon infection of mouse macrophages by parasites or bacteria.

### Silencing of Rab14 Increases Phagocytosis of the Malaria Parasite

To study if the malaria parasite modulates the expression of Rab GTPases to its own benefit, we attempted to silence one of the Rab GTPases affected, namely Rab14, and look for any changes in the phagocytosis of the parasite. We chose this Rab protein since it has been shown that phagosomes containing mycobacteria accumulate Rab14 following phagocytosis [21]. Strikingly, Rab14 silencing caused a two-fold increase in the percentage of malaria parasites phagocytosed by macrophages, as measured by flow cytometry (Fig. 4A). Moreover, we counted the number of infected cells by microscopy and confirmed the increase in phagocytosis of the malaria parasite after Rab14 silencing (Fig. S1A). Since the expression of Rab14 is not upregulated in bacterial infections, we hypothesized that this Rab would not play any role in phagocytosis of the bacteria studied. To confirm this, we measured phagocytosis by macrophages cultured with *E*. *coli*. As predicted, Rab14 silencing had no effect on the capacity of macrophages to phagocytose *E*.*coli* in any of the time points studied, as compared to control cells (Fig. 4B). Importantly, the efficiency of Rab14 silencing measured by RT-qPCR was shown to be higher than 80% (Fig. 4C). These results suggest that the increase in the expression levels of Rab proteins induced by the malaria parasite could be a mechanism of immune evasion.

### Overexpression of Rab14 Decreases Phagocytosis of the Malaria Parasite

Since we observed that silencing of Rab14 induced an increase in the phagocytosis of the malaria parasite, we hypothesized that overexpression of Rab14 would have the opposite effect. To test this, we overexpressed GFP-Rab14 in macrophages and measured phagocytosis of *P*. *berghei* and *E*. *coli* by flow cytometry (Fig. 5). The analysis of the cell population that did not overexpress Rab14 showed that around 25% of these cells had internalized malaria parasites (Fig. 5A). However, in the Rab14 overexpressing population only around 8% of the cells had internalized parasites (Fig. 5A). This effect is specific for Rab14, since the overexpression of GFP showed no difference in the phagocytosis of *P*. *berghei*, as measured by flow cytometry (Fig. 5B). Moreover, we counted the number of infected cells by microscopy and confirmed the decrease in phagocytosis of the malaria parasite in the population of cells that overexpress Rab14 (Fig. S1B), while overexpression of GFP showed no difference in phagocytosis (Fig. S1C). In contrast, phagocytosis of *E*. *coli* showed no difference between the populations overexpressing or not Rab14 or GFP (Fig. 5C and D).

### Silencing of Rab9a Increases Bacterial Phagocytosis

Having observed the striking effect on parasite phagocytosis of silencing Rab14, we decided to analyze one Rab GTPase that had its expression increased by the bacteria, namely Rab9a. This Rab protein is implicated in the late endocytic pathway, which is involved in the maturation of phagosomes into phagolysosomes. When Rab9a was silenced, we observed a significantly higher percentage of cells that had internalized *E*. *coli* as compared with siRNA control (Fig. 6A). Similar results were obtained with *Salmonella* (Fig. 6B). We also hypothesized that no effect would be observed with the malaria parasite, since the expression of this Rab was not affected in that case. Indeed, when Rab9a was silenced by siRNA no difference was detected in the phagocytosis of *P*. *berghei* as compared to siRNA control (Fig. 6C). Importantly, the efficiency of Rab9 silencing measured by RT-qPCR was shown to be higher than 80% (Fig. 6D). Thus, *E*. *coli* and *Salmonella* could inhibit phagocytosis by macrophages through the upregulation of Rab9a expression.

### Overexpression of Rab9a Decreases Bacterial Phagocytosis

Since we observed that silencing of Rab9a induced an increase in the phagocytosis of bacteria, we hypothesized that overexpression of Rab9a would have the opposite effect. To test this, we overexpressed GFP-Rab9a in macrophages and measured phagocytosis of *E*. *coli* and *Salmonella* by flow cytometry (Fig. 7). The analysis of the cell population that did not overexpress Rab9a showed that around 35% of these cells had internalized *E*. *coli* (Fig. 7A). Strikingly, in the Rab9a overexpressing population, only around 10% of the cells had internalized bacteria (Fig. 7A). This effect is specific for Rab9a, since the overexpression of GFP showed no difference in the phagocytosis of *E*. *coli* (Fig. 7B). Furthermore, similar results were observed when *Salmonella* was used (Fig. 7C and D).

### Macrophages are Attenuated for Secondary Phagocytosis of *E*. *coli*

The observation that the upregulation of Rab9a inhibits phagocytosis by macrophages suggests that this upregulation, which is induced by bacterial infection, could serve as an immune evasion strategy. This implies that the primary uptake of *E*. *coli* by macrophages would lead to the upregulation of Rab9a expression with the consequent attenuation of secondary phagocytosis. To test this hypothesis, we infected macrophages with *E*. *coli*-YFP for two hours and then superinfected the cells with *E*. *coli*-CFP. When macrophages were infected with *E*. *coli*-YFP and then superinfected, they phagocytosed significantly fewer *E*. *coli*-CFP (Fig. 8A). Moreover, we observed a 3-fold decrease in the number of CFP bacteria per cell in the case of superinfection, when compared with the infection of cells with *E*. *coli*-CFP alone (Fig. 8B). No difference was observed in the percentage of infection in cultures infected with both *E*. *coli*-YFP and *E*. *coli*-CFP (Double cultures) and cultures infected only with *E*. *coli*-CFP (Single cultures) (Fig. 8C). Therefore these results support our model that the upregulation of Rab9a expression could serve as an immune evasion mechanism.

## Discussion

Phagocytosis and the subsequent maturation of the phagosome into a lytic compartment, enabling intracellular microbial killing and degradation, constitutes the major anti-microbial defense mechanism of the innate immune system. It is known that intracellular pathogens are able to interfere with membrane trafficking pathways of the host cell, and thereby create more hospitable intracellular conditions for their survival and growth. Rab GTPases play pivotal roles in membrane trafficking [13] and intracellular pathogens can interfere with their activity and recruitment in order to establish a favourable niche [27]. However, little is known about the effect of intracellular pathogens on Rab gene expression levels. In this study, we analyzed the expression level of 23 distinct Rab GTPases on macrophages, after phagocytosis of *P*. *berghei*, *E*. *coli* and *S*. Typhimurium. To our knowledge this is the first study that comprehensively analyzes Rab expression levels upon infection by bacteria and a parasite. We found that nine Rab GTPases had their expression increased in macrophages after phagocytosis of live *P*. *berghei*. These were Rab1a, Rab1b, Rab7a, Rab10, Rab14, Rab20, Rab27a, Rab32 and Rab38. In contrast, when macrophages were stimulated with either live *E*. *coli* or *S*. Typhimurium, four Rab GTPases, namely Rab8b, Rab9a, Rab10 and Rab 20, showed an increase in their expression level. The fact that the increased expression is only observed when live parasites and bacteria are used indicates a specific effect of these microorganisms. LPS was used as a positive control since it acts as a general activator of macrophages. In response to LPS, macrophages activate different cellular pathways such as cytokine production and secretion that Rab proteins are likely to regulate. Indeed, LPS induced the upregulation of all Rab genes tested, except for Rab27a. We also attempted to analyze the protein levels of Rab9a and Rab14 upon infection. However, as with Rabs in general, the endogenous levels of these proteins were too low to be robustly detected by the antibodies available (data not shown). When we silenced Rab14 by siRNA, we observed an increase in the percentage of parasites internalized by macrophages, while no difference in phagocytosis was observed with *E*. *coli*. As predicted, the overexpression of Rab14 in macrophages induced a decrease in the phagocytosis of *P*. *berghei*, while no effect was observed with *E*. *coli*. Therefore, these results suggest a crucial role for Rab14, as a negative regulator, in the phagocytosis of the malaria parasite. Thus, it is tempting to speculate that *P*. *berghei* is able to manipulate Rab14 expression in its favor, to avoid uptake and elimination by macrophages. Importantly, our results suggest an increase in parasite uptake when Rab14 is silenced, rather than a deficiency in degradation since the effect is observed at early time points, when there is still not enough time for degradation to occur. It is conceivable that during infection, the primary uptake of *Plasmodium*-parasitized erythrocytes by macrophages would increase the levels of Rab14 and thereby inhibit subsequent phagocytic activity of the macrophages, decreasing host-mediated parasite clearance. Rab14 is involved in the maturation of early phagosomes and it has been shown to be critical in the maintenance of *M*. *tuberculosis* phagosomal arrest, leading to bacterial persistence [21]. Therefore, it seems that both malaria parasites and mycobacteria manipulate Rab14 to their own advantage, with the ultimate objective of avoiding degradation. This is in contrast with *Chlamydia trachomatis*, which was shown to require Rab14 for its development and replication [28]. We did not observe any accumulation of Rab14 around the parasite vacuole, but this does not exclude the possibility of modulation of this RabGTPase by the parasite (Fig. S2). Further studies should address the mechanism by which Rab14 plays a role in microbial infection, and in particular how an increase in Rab14 expression inhibits phagocytic uptake of erythrocytes infected with the malaria parasite.

A similar effect to that observed with Rab14 and the malaria parasite was also observed with Rab9a and both *E*. *coli* and *S*. *enterica*. Silencing of Rab9a induced an increase in the phagocytosis of both bacteria but no effect on the uptake of *P*. *berghei*. These results suggest that bacteria are able to manipulate Rab9a in order to avoid uptake by phagocytosis. Under normal physiological conditions, commensal bacteria within the gastrointestinal tract, such as *E*. *coli*, do not cause disease. However, following changes in normal homeostasis, such as what occur during inflammation and physical damage of the intestinal barrier, *E*. *coli* can translocate across this barrier, reaching circulation and causing sepsis [29], [30], [31]. To avoid this serious outcome, bacteria need to be eliminated by macrophages. In our studies, *E*. *coli* are cultured with macrophages, which effectively mimics an abnormal situation and not the normal physiological conditions, where *E*. *coli* in the gut lumen do not come in contact with macrophages. Thus, the upregulation of Rab9a could serve as a mechanism of immune evasion when *E*. *coli* escape the gut environment. Moreover, the primary uptake of *E*. *coli* by macrophages would lead to the upregulation of Rab9a expression with the consequent inhibition of phagocytosis of more bacteria. This implies that infected macrophages would be attenuated for secondary phagocytosis and, indeed, our results show this. Further studies should be performed in order to dissect the mechanism by which Rab9a plays a role in bacterial infection, through the modulation of phagocytosis.

Our results suggest that microorganisms alter the gene expression of different Rab GTPases. It is possible that the difference in the set of Rab genes whose expression is altered by the bacteria and the malaria parasite is due to the different receptors involved in the phagocytosis of the microorganisms and the different signaling cascades triggered. However, we cannot rule out that the phagocytosis of microorganisms with significantly different sizes (∼2 µm in length for *E. coli* and ∼10 µm in length for the malaria parasite) also influences the Rab genes whose expression is modulated. Future studies should clarify the mechanisms by which Rab gene expression is modulated upon infection.

Interestingly, both Rab32 and Rab38 had their expression increased in macrophages only after infection with heat-killed bacteria, whereas infection with live bacteria did not show any difference on the expression of these Rabs. This suggests that heat-killed bacteria release molecules that only in these conditions can be specifically sensed by the macrophages. Future studies should address which intracellular signalling pathways are induced by heat-killed bacteria, how this leads to Rab32 and Rab38 increased expression, and what is the physiological significance of this particular phenomenon.

In conclusion, our results show that bacteria and parasites modulate the expression of different Rab genes on macrophages upon infection and that this modulation can be done to their advantage. This is suggested by the observation that Rab14 and Rab9a, whose expression is upregulated upon infection with *P*. *berghei* and *E*. *coli* or *S*. *enterica*, respectively, are negative regulators of the phagocytosis of these microorganisms, since their silencing leads to an increase in phagocytosis. Thus, it is evident that pathogens are not passive bystanders but have evolved specific means of subverting phagocytosis and intracellular killing through different mechanisms, such as the interference with the expression of specific Rab GTPases. Further studies on the role of Rab14 and Rab9a GTPases on the phagocytosis of these microorganisms may provide targets for the development of novel therapeutic strategies.

## Supporting Information

Figure S1 **Rab14 silencing increases phagocytosis of** ***P*** **.** ***berghei*** **and Rab14 overexpression decreases phagocytosis of the parasite. (A)** Macrophages were transfected with siRNA for Rab14 or siRNA control for 48 h, and infected with GFP-*P*. *berghei*. Macrophages were transfected with GFP-Rab14 DNA **(B)** or GFP **(C)** for 8 h and then infected with RFP-*P*. *berghei*. **(A)** Columns represent the percentage of cells positive for CD11b and GFP analyzed after incubation with *P*. *berghei* (n = 300). **(B)** Columns represent the percentage of GFP-Rab14 overexpressing cells (n = 80) or cells that do not overexpress Rab14 (n = 170) with internalized RFP-*P*. *berghei*. **(C)** Columns represent the percentage of GFP overexpressing cells (n = 80) or cells that do not overexpress GFP (n = 150) with internalized RFP-*P*. *berghei*. After 15 minutes of incubation, cells were washed, chased for different time points and analyzed by microscopy. The number of cells infected with the parasite was counted at each time point as well as the total number of cells and the percentage of infection determined. Error bars indicate the standard error of the mean of 2 independent assays. Statistical significance (***p<0.001) refers to the difference between macrophages treated with siRNA for Rab14 and siRNA control or to the difference between macrophages that do not overexpress Rab14 and macrophages that overexpress Rab14.
(TIF)

Figure S2 **Rab14 localization in** ***Plasmodium*** **-infected macrophages.** Macrophages were transfected with a plasmid encoding GFP-Rab14 and infected with *P*. *berghei*-RFP. After 15 minutes of incubation, cells were washed, chased for different time points and analyzed by confocal laser scanning microscopy. Representative images are shown for 5 **(A)**, 15 **(B)** and 30 **(C)** minutes of incubation.
(TIF)

Table S1 **RT-qPCR primers.** Primer sequences used to characterize Rab GTPases by RT-qPCR.
(DOC)

Table S2 **siRNA sequences.** siRNA sequences of the siGenome Smartpool for Rab14 and Rab9a.
(DOC)
