# Supplementary figures and images for: Bacteria and Protozoa Differentially Modulate the Expression of Rab Proteins
Source: PLoS One. 2012 Jul 20;7(7):e39858. doi: 10.1371/journal.pone.0039858 (PMC3401185; doi:10.1371/journal.pone.0039858)

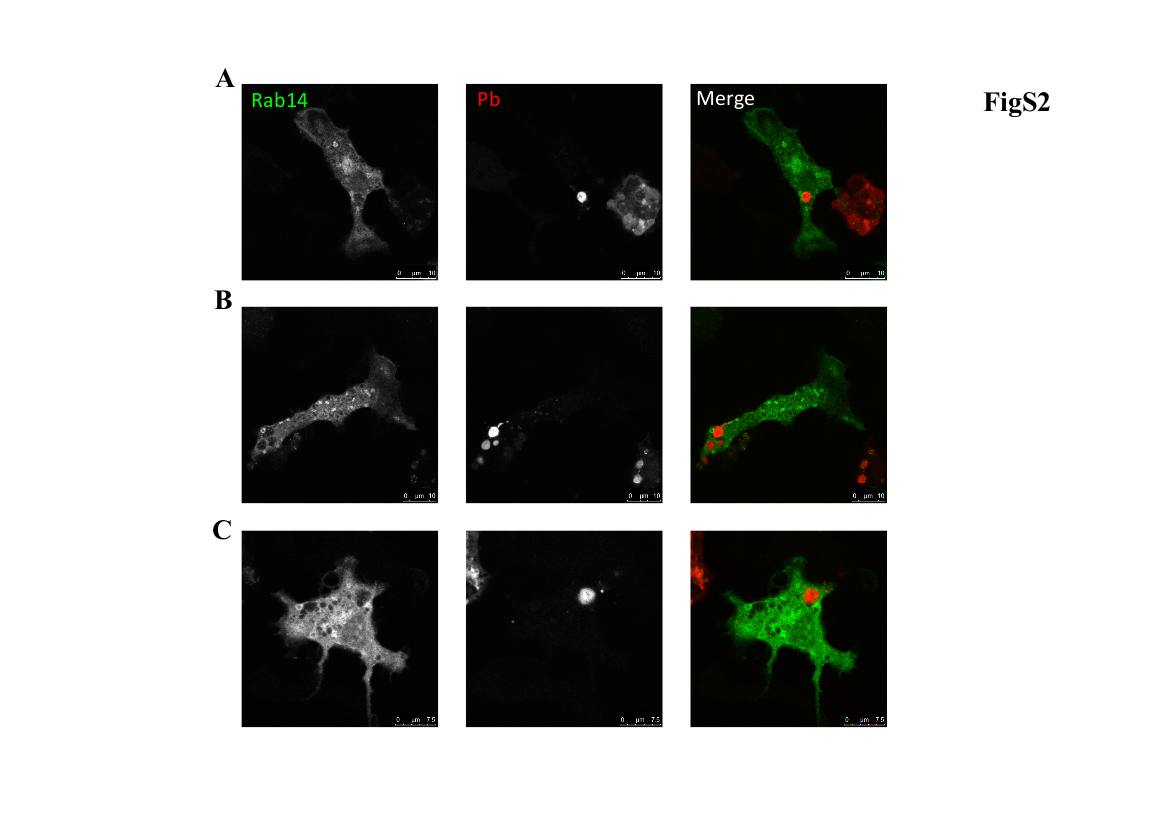

Supplement: Figure S2 — Rab14 localization in Plasmodium -infected macrophages. Macrophages were transfected with a plasmid encoding GFP-Rab14 and infected with P. berghei-RFP. After 15 minutes of incubation, cells were washed, chased for different time points and analyzed by confocal laser scanning microscopy. Representative images are shown for 5 (A), 15 (B) and 30 (C) minutes of incubation. (TIF) [file pone.0039858.s002.tif]
