# Supplementary material for: Bacteria and Protozoa Differentially Modulate the Expression of Rab Proteins
Source: PLoS One. 2012 Jul 20;7(7):e39858. doi: 10.1371/journal.pone.0039858 (PMC3401185; doi:10.1371/journal.pone.0039858)
Supplement: Table S1 — RT-qPCR primers. Primer sequences used to characterize Rab GTPases by RT-qPCR. (DOC) [file pone.0039858.s003.doc]

**Table S1 – RT-qPCR primers.**

| **Rab** | **Forward primer** | **Tm/^o^C** | **Reverse primer** | **Tm/^o^C** | **PCR product size, bp** |
| --- | --- | --- | --- | --- | --- |
| 1a | atgtgacagatcaggagtcc | 57.3 | TGACTGCTTGACTGGAGTGC | 59.4 | 326 |
| 1b | actgaccaggagtcctacgc | 61.4 | TAGCAGCAGCCACCGCTAGC | 63.5 | 335 |
| 7a | ccccaacactttcaaaaccc | 57.3 | TGGCCCGGTCATTCTTGTCC | 61.4 | 320 |
| 8b | tgctggtctatgacatcacc | 57.3 | ACTGGTCTTCTTAGACCGGC | 59.4 | 347 |
| 9a | gtgtcgatgattcacagagc | 57.3 | AAGATGAGTTTGGCTTGGGC | 57.3 | 336 |
| 10 | gtgtatgacatcaccaacgg | 57.3 | GCAGCACTTGCTCTTCCAGC | 61.4 | 339 |
| 14 | agctgcaggtgcgctcatgg | 63.5 | TGGGGTTCACTGGTTAGCCG | 61.4 | 372 |
| 20 | caaagtggacctgacctcgg | 61.4 | CCGGATCTAGTCTGTTTGGG | 59.4 | 349 |
| 27a | gttcgacctgacaaatgagc | 57.3 | TCCTCACTTAGCTGATCCGC | 59.4 | 345 |
| 32 | atcttcccaacggcagcccc | 63.5 | TCAGCAGCACTGGGACCTGG | 63.5 | 293 |
| 38 | acgctccctaatggtaagcc | 59.4 | AGCAGCTGACAACCTTGGGC | 61.4 | 280 |

Primer sequences used to characterize Rab GTPases by RT-qPCR.
