# Supplementary material for: Bacteria and Protozoa Differentially Modulate the Expression of Rab Proteins
Source: PLoS One. 2012 Jul 20;7(7):e39858. doi: 10.1371/journal.pone.0039858 (PMC3401185; doi:10.1371/journal.pone.0039858)
Supplement: Table S2 — siRNA sequences. siRNA sequences of the siGenome Smartpool for Rab14 and Rab9a. (DOC) [file pone.0039858.s004.doc]

**Table S2 – siRNA sequences.**

| **Rab** | **Target Sequence** |
| --- | --- |
| 14 | CAACUACUCUUACAUCUUU |
| 14 | ACAGAGAGAUGUUACCUAU |
| 14 | GAGGACGGCUAACCAGUGA |
| 14 | GAAAUAAAGCAGACUUGGA |
| 9a | UCACAGAGCUUCCAGAAUU |
| 9a | GAACAGAUAUGUAACCAAU |
| 9a | CAACAAGACUGACAUAAAA |
| 9a | AAACUCAUCUUGCUGUUGA |

siRNA sequences of the siGenome Smartpool for Rab14 and Rab9a.
